# Video Texture Synthesis Based on Flow-Like Stylization Painting

**DOI:** 10.1155/2014/689496

**Published:** 2014-07-15

**Authors:** Qian Wenhua, Xu Dan, Yue Kun, Guan Zheng

**Affiliations:** Department of Computer Science and Engineering, School of Information Science and Engineering, Yunnan University, Kunming 650091, China

## Abstract

The paper presents an NP-video rendering system based on natural phenomena. It provides a simple nonphotorealistic video synthesis system in which user can obtain a flow-like stylization painting and infinite video scene. Firstly, based on anisotropic Kuwahara filtering in conjunction with line integral convolution, the phenomena video scene can be rendered to flow-like stylization painting. Secondly, the methods of frame division, patches synthesis, will be used to synthesize infinite playing video. According to selection examples from different natural video texture, our system can generate stylized of flow-like and infinite video scenes. The visual discontinuities between neighbor frames are decreased, and we also preserve feature and details of frames. This rendering system is easy and simple to implement.

## 1. Introduction

Most natural phenomena can use image or video to demonstrate directly. However, both natural and artificial phenomena cannot adequately be captured by a single static photo or image. On the other hand, though video is considered as the best media to show natural scene, it also has some shortcomings, such as if video scene should be stored on a computer or other storage devices, we always apply finite duration video clip. Therefore, video needs a beginning, a processing, and the ending frame. Further, though video captures the time-varying behavior of the phenomenon, it lacks the “timeless” quality of the photograph or image. In addition, it needs too much storage space.

In order to solve the above problems, Schödl and Essa proposed a new type of medium called “video texture” [1]. Video texture has qualities between photograph and video. This medium provides a continuous, infinitely varying stream of video images, and it can show an arbitrary length of video. The natural short video can be captured to synthesize infinite scene based on the technique of video texture synthesis. Then video texture works well for motions not only repetitive but also quasi-repetitive, such as swaying trees, waterfalls, and flickering flames. So, video texture is the best media to represent natural phenomena of our nature. Because of its wide application in the entertainment and industry and so forth, computer-based phenomena video texture becomes hot topic.

Generally, video texture may be regarded as expansion of image not only in the time domain but also in natural phenomena simulation. Schödl and Essa also employed video texture technique to animation. They used L2 distance to measure similarity between neighbor frames and maintained playing continuity among video frames. The proposed method has realized highly third dimension scenery animation successfully. Based on image cutting and synthesizing technique, Kuwahara improved video texture synthesis method. To ensure continuity, the input frames were divided into some parts, and then they would be synthesized to video texture [2]. Bhat et al. put forward an editing nature phenomena method to generate the final video texture. Their method first analyzed the motion of texture particles and segmented frames into patches, then they reorganized the patches to reduce error dither [3]. Their method can merge an arbitrary length video based on natural scene. Agarwala et al. described an automatic method for taking the output of a single panning video camera and creating a panoramic video texture [4]. Their method applied a dynamic programming step through hierarchical min-cut optimization process to improve synthesis speed.

Though there are so many video synthesis methods to synthesize natural video phenomena, the synthesis results are traditionally striven photorealism scenes. That is to say photorealism effects can not produce artistic sensation. Sometimes, people want to enjoy artistic video with cartoon, oil, and water painting effects. In addition, photorealistic images often include more details than necessary to communicate intended information. It is known that nonphotorealistic rendering (NPR) is the subject of intense debate for representative methods of artistic style creation. Artistic expression can often convey a specific mood which is difficult to imbue in a photorealistic scene. NPR also can focus viewer's attention on the important information while downplaying extraneous or unimportant features. Additionally, a NPR look is often more engaging than the traditional, photorealism computer graphic rendering [5]. So, if video texture can be rendered to some nonphotorealistic effects, we can obtain artistic video with infinite playing scene.

Some NPR methods address the computer generated abstract stylization artistic effects, and this process fit perceptual task performance and evaluation. Decaudin applied modeling tools to generate abstract effect from natural scene [6], and his method kept details very well and shadow stayed coherent. Based on mean-shift, DeCarlo and Santella extended three-dimensional video volumes to automatically generate abstract artistic [7]. Particularly in the presence of occlusions and camera movement, contour tracking required substantial user correction of the segmentation results.

This paper presents an improved method to synthesize phenomena video with flow-like abstract painting. The new NP-video model can convert natural phenomena to a flow-like stylization video fast, and we also can achieve the infinite video texture easily. The amalgamations of the NPR and video texture synthesis can make the user enjoy artistic phenomena work, looking on the original phenomena in different artistic style, immersing vision and spirit into the artistic kingdom, and avoid some critical problems in photorealism systems.

## 2. NP-Video Phenomena Model System

**Figure 1:**
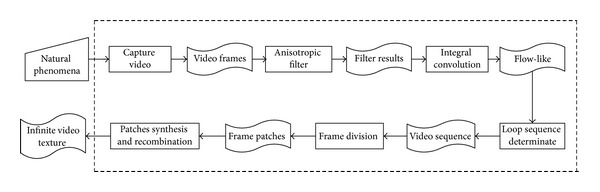
NP-video phenomena of our system.

We will introduce the basic architecture of our NP-video phenomena model in this section. As shown in Figure 1, our system proceeds in two steps. (1) Flow-like effects rendering: the natural phenomena video should be rendered to NPR artistic scene. (2) Video texture synthesis: the phenomena with flow-like effect will be synthesized to infinite playing video. We define the final play sequence of video texture to eliminate visual discontinuities. In addition, frames dividing and recombination method is used to arrive coherent playing frames. Our architecture supports a common NPR technique merging to video texture, and the final NP-video can give user flexibility and enjoyment.

## 3. Flow-Like Artistic Style Simulating

Many arts create flow-like artistic work, and people always enjoy this work. Van Gogh and Munch emphasized flow-like structures and directional features in their paintings. The paintings are so famous because they are harmonic, interesting, and pleasant. Directional coherence and flow structure in this artistic work can help salience region features and boundaries. It also helps to evoke viewer's imagination. So, before synthesizing video texture, we transfer the input phenomena to flow-like art first, and our method provides a versatile NPR flow-like rendering interface.

As same as the abstract effects, flow-like stylization artistic work can generate using edge-preserving filter method. Bilateral filter [8] and mean-shift method [9] are very famous examples of edge-preserving filter. Kang et al. improved the filter shapes, which is based on the vector field derived from the salient image features [10]. However, because edge-preserving filter cannot obtain good result with weak vector, this method may lose some details such as weak edge and structure. Kuwahara filter provides an overall stereoscopic painting visual and maintains a uniform level of abstraction across the image [11]. Unfortunately, Kuwahara filter can result in clustering artifacts during filter process. Papari et al. introduced a new weighting filter window, and the shape of the filter window can be changed based on local feature directions [12]. Kyprianidis also generated an abstract effect along the local feature directions [13], but the stylized surface of their methods cannot preserve both spatial and temporal coherence. Like bilateral filter and Kuwahara filter, Kang and Lee used Shock filter and mean curvature flow to generate abstraction effect, but the boundaries are not salient and simplified [14]. Recently, Kim et al. put forward bristle maps to generate aggregation, abstraction, and stylization of spatiotemporal data [15]. Isenberg explored visual map technique for generating of stylized renderings of 2D map data [16]. His method can render different stylization artistic work.

Though there are many techniques that can be used to obtain flow-like artistic work, these methods should be improved to adopt phenomena video. After capturing phenomena video, we will resolve this video and generate flow-like stylization painting of every frame. Our method begins with the anisotropic Kuwahara filtering. Firstly, eigenvalues and eigenvectors can be used to calculate structure tensor. Secondly, local orientation and anisotropy structure are used to guide Kuwahara filter process. Then the flow-like effects can be generated using improved line integral convolution.

### 3.1. Local Orientation Calculate

Brox et al. obtained anisotropy through calculating eigenvalues and eigenvectors of structure tensor [17]. We use Brox's method to calculate structure tensor first. Let *G*_*σ*,*x*_ and *G*_*σ*,*y*_ be the spatial derivatives in *x*- and *y*-direction and let *f* be the input frame with standard deviation *σ*; structure tensor can be calculated as

(1) $$\begin{array}{c} G_{\sigma ,y}=\frac{1}{2\pi \sigma^{2}}e^{-{\left(y/\sqrt{2}\sigma \right)}^{2}},\;\;G_{\sigma ,x}=\frac{1}{2\pi \sigma^{2}}e^{-{\left(x/\sqrt{2}\sigma \right)}^{2}}. \end{array}$$

The pixel's gradient vector is **G** = (*G*_*x*_, *G*_*y*_)^*T*^. Let ∗ denote convolution operation; the partial derivatives of *f* can be calculated:

(2) $$\begin{array}{c} fx=G_{\sigma ,x}*f,\;\;fy=G_{\sigma ,y}*f. \end{array}$$

The structure tensor of each video frame **f** can be defined as

(3) $$\begin{array}{c} T_{x,y}=G\times G^{T}=\left[\begin{array}{cc} f_{x}^{2} & f_{x}f_{y}\\ f_{x}f_{y} & f_{y}^{2} \end{array}\right]=\left[\begin{array}{cc} T_{11} & T_{12}\\ T_{21} & T_{22} \end{array}\right]. \end{array}$$

Let *d*_1_ and *d*_2_ be the minimum and the maximum eigenvalue of **T**, which represents gray scale variation degree of vector **v**_1_ and **v**_2_

(4) gσ,γ,θ=d1+d22πσ2exp⁡[−x2+r2y22θ2/(d1+d2)],

where parameter *σ* denotes filter kernel of Gauss function variance, *r* denotes filter radius which is calculated using *d*_1_/*d*_2_. If *d*_1_ and *d*_2_ are close, this filter field tends to be circular. The parameter *θ* represents edge direction of pixel (*x*, *y*), which reflects curve direction of eigenvalue. Then anisotropic structure tensor can be expressed as **T**′:

(5) $$\begin{array}{c} T^{\prime }=g_{\sigma ,\gamma ,\theta }\times T=\left[\begin{array}{cc} g_{\sigma ,\gamma ,\theta }\times T_{11} & g_{\sigma ,\gamma ,\theta }\times T_{12}\\ g_{\sigma ,\gamma ,\theta }\times T_{21} & g_{\sigma ,\gamma ,\theta }\times T_{22} \end{array}\right]. \end{array}$$

Because the local vector field maybe discontinuous, we use Gaussian filter technique to smooth this vector field, and eigenvalues of structure tensor are given:

(6) $$\begin{array}{c} Z_{1,2}=\frac{T_{11}^{\prime }+T_{22}^{\prime }\pm \sqrt{{\left(T_{11}^{\prime }-T_{22}^{\prime }\right)}^{2}+4T_{12}^{\prime }T_{21}^{\prime }}}{2}. \end{array}$$

Then, the eigenvector in local curve direction is calculated as

(7) $$\begin{array}{c} t=\left(\begin{array}{c} Z_{1}-T_{11}^{\prime }\\ -T_{12}^{\prime } \end{array}\right). \end{array}$$

The local orientation is *φ* = arg⁡*t*, and we calculate anisotropy based on Yang's method [18]:

(8) $$\begin{array}{c} A=\frac{Z_{1}-Z_{2}}{Z_{1}+Z_{2}}, \end{array}$$

where parameter *A* ranges between 0 and 1, which denotes isotropic and anisotropic regions, respectively.

### 3.2. Anisotropic Filter

Because pixel (*i*, *j*) of filter result is determined by the spatial distance from the center pixel (*x*, *y*), as well as its relative intensity difference, the filtering output *Q* at pixel (*i*, *j*) is calculated:

(9) $$\begin{array}{c} Q_{i,j}\left(x,y\right)=\frac{\sum_{x,y\in \Omega }a_{i,j}\left(x,y\right)m_{i,j}\left(x,y\right)}{\sum_{x,y\in \Omega }a_{i,j}\left(x,y\right)}, \end{array}$$

where parameter Ω denotes filter spatial space. *G*_*i*,*j*_ is the local intensity or color similarity with weighted *w*_*i*,*j*_, and *a*_*i*,*j*_ denotes the squared standard deviations, *m* and *a* can be defined:

(10) $$\begin{array}{c} m_{i,j}\left(x,y\right)=\frac{1}{k}\underset{x,y\in \Omega }{\sum }f_{i,j}\left(x,y\right)w_{i,j}G_{i,j}\left(x,y\right),\\ a_{i,j}\left(x,y\right)=\frac{1}{k}\sqrt{\underset{x,y\in \Omega }{\sum }f_{i,j}^{2}\left(x,y\right)w_{i,j}G_{i,j}\left(x,y\right)-m_{i,j}^{2}},\\ k=\underset{x,y\in \Omega }{\sum }w_{i,j}G_{i,j}\left(x,y\right). \end{array}$$

**Figure 2:**
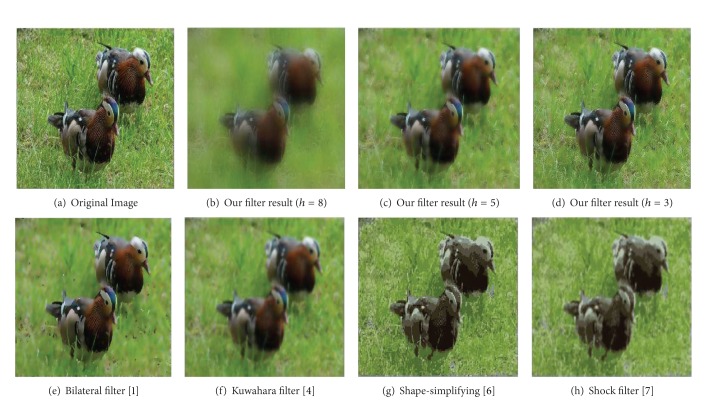
Comparison of different filter.

**Figure 3:**
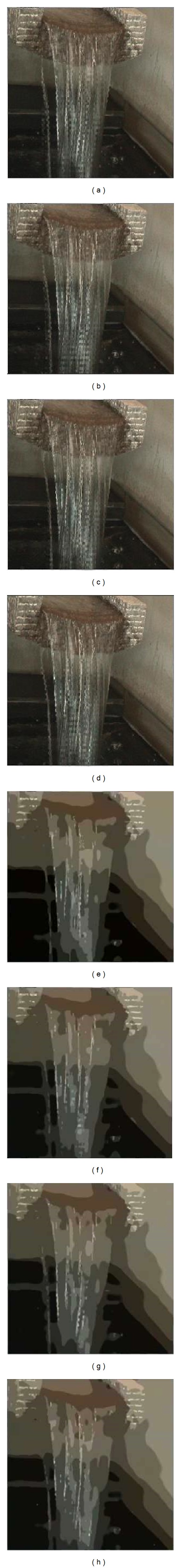
Video texture filter of blowing water.

Let *φ* be the local orientation, *A* be anisotropy, and *Rφ* be matrix defining a rotation, the filter spatial space Ω is

(11) $$\begin{array}{c} \Omega_{i,j}=\left\{\left(x,y\right)\in R^{2}:\text{abs}\left(SR_{-\varphi }\left(x,y\right)\right)\leq h\right\}, \end{array}$$

where parameter *h* = 2*σ*_*r*_. Parameter *S* is coefficient to adjust anisotropy *A*. So, *SR*_−*φ*_ is a linear coordinate transform which maps Ω to a disc with radius *h*. This anisotropic filter ensures that if local region has lower standard deviation, the more weight value is given during filter process.  Figure 2 shows our filter results.  Figure 2(a) is input image, and Figures 2(b) and 2(d) are our results with different *h*. If *h* is smaller, more details will be retained. Figures 2(e) and 2(h) are filter results with other methods.  Figure 3 also shows another result with blowing water. Figures 3(a) and 3(d) are different frame of natural texture, and Figures 3(e) and 3(h) are filter results. From these results, we can find that our method can maintain more details and features, such as edge, color, and local structure. Our method also eliminates artifacts of shape-simplifying and Shock filter method.

### 3.3. Integral Convolution

We benefit from our efficient stylization painting not only in anisotropic filtering, but also with flow-like effect. Papari and Petkov applied integral convolution technique to produce nonphotorealistic rendering glass patterns texture effect [19]. We refer their integral convolution method to generate final artistic work. During convolution process, our method calculates a local stream line which moves out from the positive and negative directions. Let *I*_out_ be flow-like output image, and let *σ*(*s*) be a stream line with length of *L*. Parameter *p*_0_ denotes each pixel in *σ*(*s*). Let *s*_0_ be current pixel; we can obtain *p*_0_ = *σ*(*s*_0_). Parameter *k*(*s*) denotes convolution kernel, and we utilize Hanning window function in this paper. *I*(*σ*(*s*)) denotes all the pixels in this line, the convolution implement is defined as follows [20]:

(12) $$\begin{array}{c} I_{\text{out}}\left(p_{0}\right)=\overset{s_{0}+L/2}{\underset{s_{0}-L/2}{\int }}k\left(s-s_{0}\right)I\left(\sigma \left(s\right)\right)ds, \end{array}$$

where parameter *L* denotes convolution length. If *L* is small, an insufficient amount of filtering flow occurs. However, too large *L* will result in close convolution value and regular output. Because variance can well reflect local statistic information, it can be used to calculate local convolution length automatically:

(13) $$\begin{array}{c} L_{\left(x,y\right)}=L_{\max}-\frac{L_{\max}-L_{\min}}{\sigma_{\max}-\sigma_{\min}}\left(\sigma_{\left(x,y\right)}-\sigma_{\min}\right), \end{array}$$

where *L*_max⁡_ and *L*_min⁡_ are the largest and the smallest integral length which can be assigned. Parameter *σ* is variance value of local area. Based on anisotropic filtering and integral convolution, input video can be rendered to flow-like effects. Next we will synthesize nonphotorealistic video texture to infinite video scene.

## 4. Phenomena Video Texture Synthesis

After phenomena video is rendered to flow-like effects, we will synthesize video texture clip to infinite video scene through video texture synthesis method. There are two significant challenges for video texture synthesis. (1) Because infinite video scene is generated through playing period video sequence repeat, the end frame in one sequence has large difference with the beginning frame in the next loop sequence. So, there are visual breaks between different loop arrays. (2) During video texture playing, visual discontinuities exist in different frames in one sequence. When frames dispose to nonphotorealistic artistic work, the discontinuities will be more prominent. So, we will determinate loop sequence of input video frames first. Then, methods of frame division and recombination, patches synthesis will be applied to eliminate visual discontinuities.

### 4.1. Loop Sequence Determinate

Many natural phenomena present playing cycle in video texture. If we play video cycle of natural phenomena repeat, people can enjoy infinite scene, and visual discontinuities will be reduced. So, we should find the loop cycle of a certain video texture. We use Schödl's method of L2 distance to measure the similarity among different video frames [1].

Because human's vision is sensitive to the change of luminance in frames, during the process of L2 distance calculation, we transfer the color space of frame from RGB to YCbCr. So L2 distance can be calculated only in luminance channel Y. If *N*_*i*_, *N*_*j*_ denote two different frames in one video texture, similarity can be acquired as

(14) $$\begin{array}{c} E\left(N_{i},N_{j}\right)=\underset{p\in n_{i,\;}p^{\prime }\in n_{j}}{\sum }{\left[I_{i}\left(p\right)-I_{j}\left(p^{\prime }\right)\right]}^{2}, \end{array}$$

where *I*_*i*_, *I*_*j*_ are luminance information of *N*_*i*_ and *N*_*j*_. Parameters *p*, *p*′ represent pixel positions of *N*_*i*_ and *N*_*j*_. So, if *E*(*N*_*i*_, *N*_*j*_) is smaller than the threshold *k*, frames *N*_*i*_ and *N*_*j*_ can be regarded as the same loop sequence. That is to say, *N*_*j*_ and *N*_*i*_ are neighbor frames. If *n* denotes frame number of input natural video texture and *m* denotes each frame in *n*; threshold *k* can be calculated:

(15) $$\begin{array}{c} k=\frac{1}{n-1}\overset{n-1}{\underset{m=1}{\sum }}E_{m,m+1}. \end{array}$$

Based on similarity, video texture can be divided into different sequences. Because L2 distance of neighbor frames in one sequence is small, we consider using different sequences to combine final infinite video scene. If L2 distance satisfies formula (16), then there only exists one sequence, and this sequence of input video texture has *n* frames:

(16) $$\begin{array}{c} E_{1,2}<E_{1,3}<\cdots <E_{1,m}<E_{1,m+1}>\cdots >E_{1,n}<E_{1,n+1}. \end{array}$$

**Figure 4:**
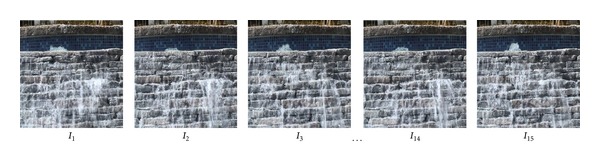
Natural input video texture.

Figure 4 shows one natural video texture of stream water. There are fifteen frames *I*_1_ to *I*_15_ in this video, and we can obtain similarity in Table 1 using formula (14).

From Table 1, L2 distance satisfied *E*_1,2_ < *E*_1,3_ < ⋯<*E*_1,7_ > *E*_1,8_ > ⋯>*E*_1,14_ < *E*_1,15_. Then frames *I*_1_ to *I*_14_ belong to the one sequence, and fourteen is the cycle length of this video. If we want to obtain infinite video texture, frame *I*_1_ should be played when frame *I*_14_ is ended, and these fourteen frames should play repeatedly.

### 4.2. Frame Division and Recombination

**Figure 5:**
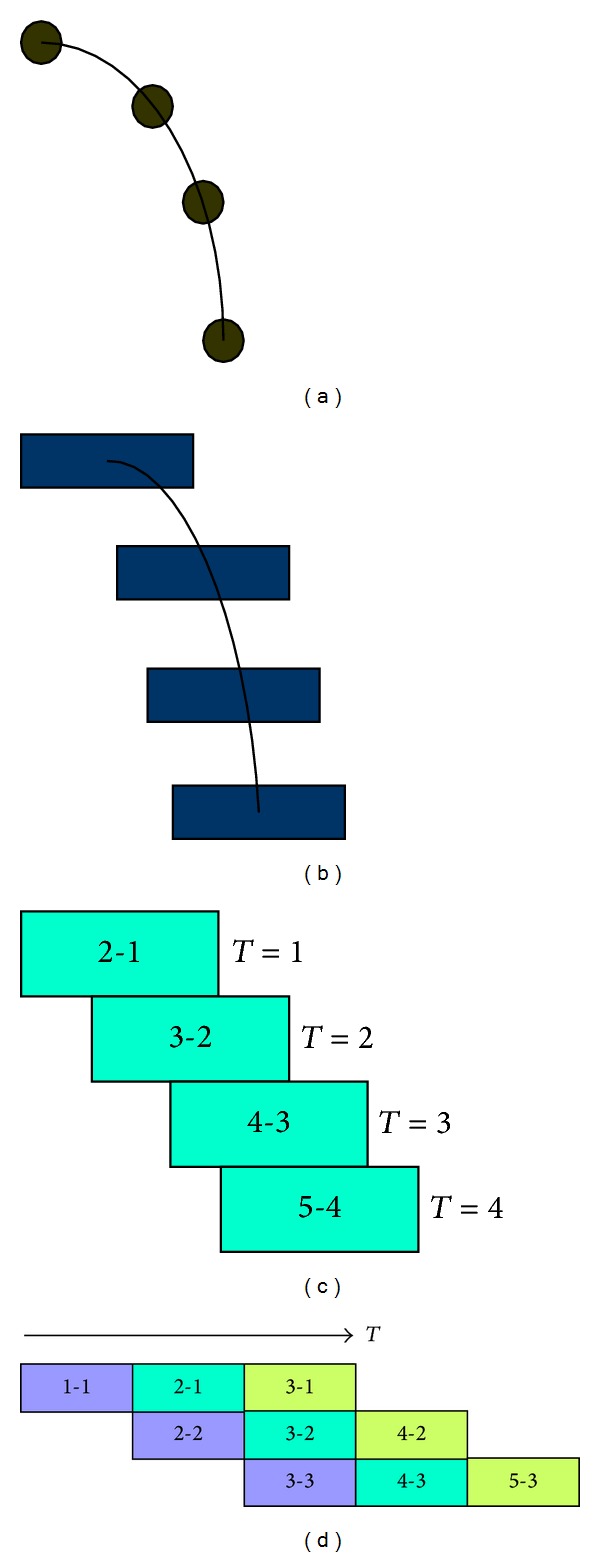
Natural phenomena moving like particle.

Though L2 distance is smaller than threshold in one sequence, when playing video sequence repeatedly, there also exists visual discontinuity between neighbor frames, such as ending and beginning frames. Therefore, we utilize Bhat's method to eliminate these discontinuities. Most natural phenomena video texture is similar particle moving, such as waterfall, fountain, flame, and stream water.  Figure 5(a) shows a particle moving procedure along a flow line, and we can find that a patch texture of one frame in Figure 5(b) will shift to neighbor frame in next playing moment.

One frame in video texture can be divided into some texture patches, such as Figure 5(c). If the sequence of input video is four, then each frame should be divided into four texture parts, and each patch will shift along flow line like Figure 5(a). The patch will move to different position in neighbor frames. For example, when *T* = 3, the patch moves to the third part in the fourth frame, whose beginning is the first part in the second frame. If frames are divided into three parts, Figure 5(d) shows the result after frames recombined together, and the same color stands for the same texture patch. We can find that the same patch is in different frames when this video scene is playing.

Table 2 shows original playing sequence without frame dividing and recombination. The different column expresses different playing video frames. When this video texture is playing repeatedly, there is a visual discontinuity between the end frame of front cycle and the beginning frame of next cycle. If sequence length of this input video texture is three, then we divide each frame into three texture parts. After using frames recombination method, we obtain new group frames like Table 3. From Table 3, we can find that the discontinuity is scattered to different frames and forms a ladder-shaped discontinuity. Because this technique disperses visual dither to the whole playing sequence, visual break between different loop arrays will be reduced.

The method of frames division and recombination is suitable to natural phenomena moving downwards, such as rivers, waterfalls, and fallen leaves. If input video texture moves upwards, such as fountains or flames, the frames should be divided into patches and recombination retroflex like Table 3. However, if a natural phenomenon is complicated and its sequence is more than ten, it is impossible to divide every frame into ten patches. Then, if video texture's sequence is more than appointed threshold, each frame of this video can be divided using this threshold.

### 4.3. Patches Synthesis Method

Though visual discontinuity can be removed applying frame division and recombination, some distinct boundaries will be produced between different patches in one frame. Texture synthesis method can be used to eliminate these boundaries.

**Figure 6:**
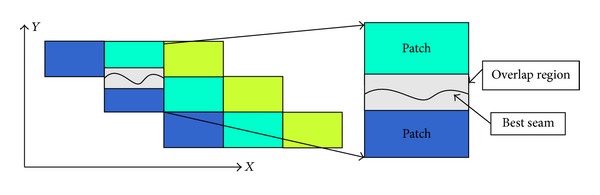
Overlap regions between patches.

Texture synthesis method can be used to synthesize texture sample to an unlimited size image. Efros and Freeman proposed a texture quilting method to synthesize surprisingly good results for a wide range of textures [21]. Efros and Freeman chose some blocks from the sample texture to fill in the final texture image. The blocks satisfy the overlap constraints within some error tolerance. As shown in Figure 6, there have been some overlap regions between neighbor blocks.

To eliminate discontinuity between overlap regions, based on the error cost path in overlap region, Efros found the best seam line to synthesize neighbor blocks. Hence, when frames of input video texture are divided into patches, we apply Efros' method to recombine these patches. Let *E*_(*i*,*j*)_ be error cost function and *M* width of overlap region. *E*_color_ denotes distance error of color, *E*_geometry_ denotes structure error, and the minimum error boundary can be calculated as

(17) $$\begin{array}{c} E\left(i,j\right)=\min\overset{M}{\underset{k=1}{\sum }}\left(E_{\text{color}}{\left(i,j\right)}^{2}+E_{\text{geometry}}\left(i,j\right)\right). \end{array}$$

**Figure 7:**
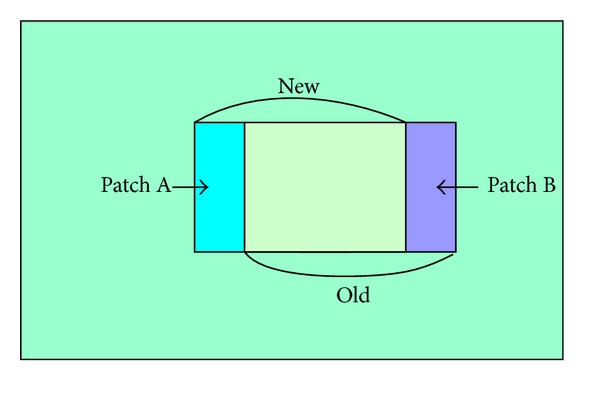
The slide of matching window.

Figure 7 shows the slide window when we calculate *E*(*i*, *j*). Let *E*_pre_ be error cost of previous slider window and *e*_old_ error cost of region A; *e*_new_ is error cost of region B. Equation (18) is used to calculate the error cost between* New* and* Old* windows, and this method can optimize synthesis process between different patches:

(18) E(N1,N2)=Epre(N1′,N2′) −eold(N1′,N2′)+enew(N1,N2).

## 5. Experiment Results

**Figure 8:**
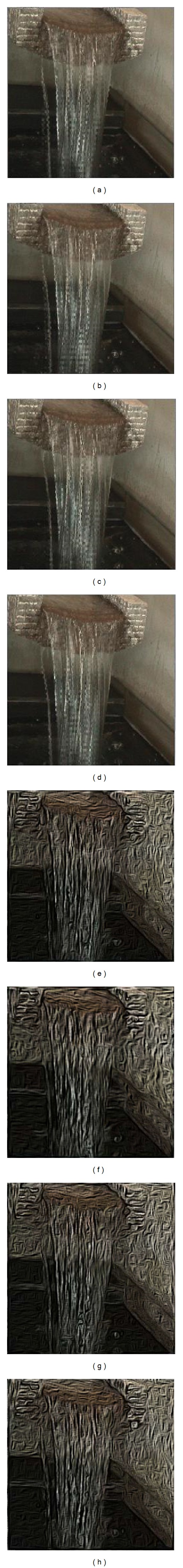
Video texture of blowing water.

To verify the feasibility and effectiveness of our proposed methods in this paper, we implemented the relevant algorithms and carried out our corresponding experiments. Figures 7 and 8 show the experimental results of video texture about blowing water and fountain scene. Our system works on Microsoft Visual C and the OpenGL texture mapping routines.

**Figure 9:**
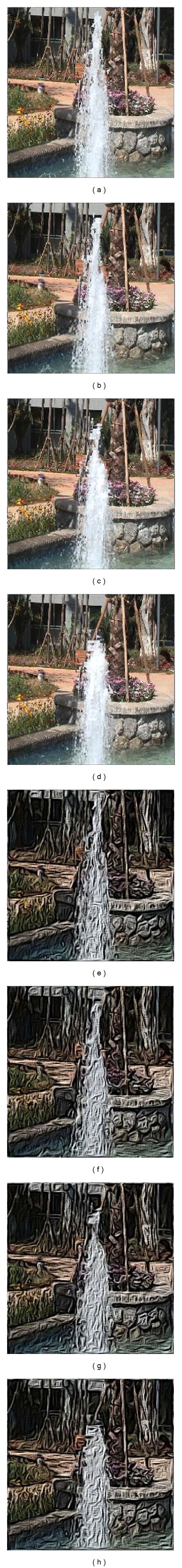
Video texture of fountain.

Figure 8 shows blowing water video scene, and this input video has seventeen frames.  Figure 9 shows fountain video scene, and this input video has fifty-eight frames. Figures 8(a) and 8(d) and Figures 9(a) and 9(d) are input frames from natural texture samples. The size of input frames is 256∗256. Based on L2 distance calculation, we choose eight frames as loop sequence from blowing water and choose eighteen frames as loop sequence from fountain video scene. Figures 8(e) and 8(h) and Figures 9(e) and 9(h) show final frames with flow-like stylization effects based on our nonphotorealistic rendering and video texture synthesis methods. We can obtain the infinite video scenes playing loop sequence repeat, and this infinite video scenes has flow-like artistic effect. We also can conclude that the experimental results are realistic and they all have some esthetics.

## 6. Conclusions

In this paper, we have presented an NP-video texture synthesis system based on natural phenomena. Based on a simple and effective anisotropic filtering and integral convolution, flow-like stylization video effects can be generated. In addition, through loop sequence determination, frame division, and recombination, our algorithm removes visual discontinuities between different frames during video texture synthesis process. The experiment results indicate that our algorithm is easy to synthesize infinite video scene with nonphotorealistic artistic work.

An obvious limitation of our method is that the synthesis speed should be improved. Some strategies should be used to protect highly important area in the frames. We may further extend the GPU implementation of our algorithm to process video in real time.

## Figures and Tables

**Table 1:** L2 distance between the first frame and others.

	2	3	4	5	6	7	8	9	10	11	12	13	14	15
1	4776	6206	7069	7124	7125	7454	7453	7114	6952	6117	5822	5549	5049	5305

**Table 2:** Original frame loop sequence.

$\overset{\;\;\;\;\;\;\;\;\;\;\;\;\;\;\;\;\;\;\;\;\;\;}{\to }T$									
1-1	2-1	3-1	4-1	5-1	1-1	2-1	3-1	4-1	5-1
1-2	2-2	3-2	4-2	5-2	1-2	2-2	3-2	4-2	5-2
1-3	2-3	3-3	4-3	5-3	1-3	2-3	3-3	4-3	5-3

**Table 3:** Frame division and recombination.

$\overset{\;\;\;\;\;\;\;\;\;\;\;\;\;\;\;\;\;\;\;\;\;\;}{\to }T$								
1-1	2-1	3-1	1-1	2-1	3-1	1-1	2-1	3-1
1-2	2-2	3-2	4-2	2-2	3-2	4-2	2-2	3-2
1-3	2-3	3-3	4-3	5-3	3-3	4-3	5-3	3-3
